# Influence of symptom burden on physical activity among patients with atrial fibrillation: The chain-mediating roles of exercise sensitivity and kinesiophobia

**DOI:** 10.1371/journal.pone.0352864

**Published:** 2026-06-30

**Authors:** JianMei Wu, ChunYun Pu, Li Zhang

**Affiliations:** 1 Nursing Department, The First Affiliated Hospital of Chongqing Medical University, Chongqing, China; 2 School of Nursing, Chongqing Medical University, Chongqing, China; 3 Department of Cardiology, The First Affiliated Hospital of Chongqing Medical University, Chongqing, China; Universitatsklinikum Schleswig Holstein Campus Lubeck, GERMANY

## Abstract

**Objective:**

Grounded in an integrated fear-avoidance and cognitive-behavioural framework, this study aimed to examine the independent and sequential mediating roles of exercise sensitivity and kinesiophobia in the relationship between symptom burden and physical activity in patients with atrial fibrillation.

**Methods:**

This cross-sectional study recruited 533 patients with atrial fibrillation from five tertiary hospitals in Chongqing, China, between April and October 2024 using convenience sampling. Symptom burden was assessed using the University of Toronto Atrial Fibrillation Severity Scale (symptom subscale), exercise sensitivity with the Exercise Sensitivity Questionnaire, kinesiophobia with the Tampa Scale for Kinesiophobia Heart, and physical activity with the International Physical Activity Questionnaire-Short Form. Data were analysed using SPSS 25.0, and chain-mediation analysis was performed with the PROCESS macro (Model 6, 5000 bootstrap resamples).

**Results:**

Symptom burden was significantly and directly associated with reduced physical activity (accounting for 37.00% of the total effect). Three significant indirect pathways were identified: through exercise sensitivity alone (27.60% of the total effect), through kinesiophobia alone (16.17%), and sequentially through exercise sensitivity and kinesiophobia (19.22%). The total indirect effect accounted for 62.99% of the total effect; the 95% bootstrap confidence intervals for all indirect effects did not contain zero.

**Conclusion:**

Exercise sensitivity and kinesiophobia were identified as statistically significant independent and sequential mediators between symptom burden and physical activity in patients with atrial fibrillation. These results suggest that routine screening and interventions addressing these psychological factors may be beneficial in atrial fibrillation management.

## 1. Introduction

Atrial fibrillation (AF) is the most common sustained cardiac arrhythmia globally, with a lifetime risk of 30–33% [1]. It is strongly associated with a substantially elevated risk of stroke, heart failure, and all-cause mortality, posing a major public health challenge worldwide [2,3]. Patients with AF frequently experience symptoms such as palpitations, fatigue, and dyspnoea. These symptoms, alongside serious complications including stroke and heart failure, markedly reduce quality of life [4]. Strong evidence demonstrates that achieving 150 minutes of moderate-intensity physical activity per week can effectively alleviate AF-related symptoms, improve exercise tolerance and quality of life [5], and reduce the risk of heart failure and mortality [6]. Therefore, clinical guidelines strongly recommend that patients with AF engage in at least 150 minutes of moderate-intensity activity weekly [7,8]. In practice, however, up to 67.4% of AF patients are physically inactive, defined as engaging in less than 150 minutes of moderate-intensity or 75 minutes of vigorous-intensity activity per week [9]. This inactivity may not only worsen symptom burden but also increase the risk of death, heart failure, and stroke in this population [10,11]. Therefore, elucidating the mechanisms underlying physical inactivity in patients with AF is crucial for developing effective strategies to improve clinical outcomes.

It is well established that somatic symptoms directly limit physical activity [12,13]. In patients with AF, symptoms such as palpitations, dyspnoea, and reduced exercise tolerance are associated with a significant decline in physical activity [14]. Furthermore, evidence indicates that somatic symptoms could impede physical activity through psychological mediators, such as catastrophic thinking and fear, as seen in chronic conditions like osteoarthritis and post-renal transplantation [15,16]. These findings suggest that the adverse effect of symptom burden on physical activity in AF involves complex psychological mediating mechanisms. Elucidating these mechanisms is essential for a deeper understanding of physical activity behaviour and for designing targeted interventions.

Exercise sensitivity—defined as the fear of bodily sensations arising during physical exertion [17] —may represent a critical psychological factor [18]. AF-related symptoms can heighten patients’ vigilance towards internal bodily signals [19], predisposing them to misinterpret normal physiological responses during exercise (e.g., increased heart rate and respiratory rate) as signs of AF exacerbation or onset. This catastrophic cognitive bias may foster a persistent fear of exercise-associated sensations [18], thereby leading to activity avoidance [17]. We therefore hypothesise that exercise sensitivity mediates the relationship between symptom burden and physical activity.

Another significant psychological barrier is kinesiophobia—an excessive and irrational fear of physical activity or exercise stemming from a fear of injury or re-injury [20]. It is a key psychological factor that constrains physical activity in various chronic disease populations [21,22]. Kinesiophobia not only hampers the initiation and maintenance of exercise [23] but is also positively associated with symptom burden [24]. Thus, it is hypothesised that kinesiophobia mediates the relationship between symptom burden and physical activity.

A recent study in patients with chronic coronary syndrome demonstrated that exercise sensitivity is associated with both kinesiophobia and reduced physical activity [25], providing preliminary evidence for the interconnections among these constructs in cardiovascular populations. However, whether exercise sensitivity and kinesiophobia function sequentially as mediators linking symptom burden to physical activity has not yet been examined. Addressing this question requires a theoretical framework that can explain how symptoms translate into activity avoidance through psychological mechanisms. The Fear-Avoidance Model provides such a framework. Although originally developed for chronic pain, the Fear-Avoidance Model describes a cognitive-behavioural sequence—catastrophic misinterpretation of bodily sensations leading to fear and subsequent avoidance—that is not specific to nociception. A recent theoretical reconceptualisation has reframed the model as a framework of “embodied threat prediction,” in which any bodily sensation perceived as threatening can activate the fear-avoidance cycle [26]. When applying this model to cardiac populations, Bäck et al. explicitly replaced “fear of pain” with “fear of a heart incident” [27], and the model has since been directly applied to patients with AF [28].

In AF, normal physiological responses to physical activity—such as increased heart rate and respiratory rate—closely resemble the symptoms of AF, and may therefore be misinterpreted by patients as signals of AF onset or cardiac decompensation, engaging the same catastrophic appraisal process. In chronic pain, patients can often identify specific triggers and selectively avoid particular movements. In AF, however, symptoms often arise without warning, which may foster a state of diffuse hypervigilance in which patients may avoid exercise not because it has consistently triggered symptoms but because it could.

To systematically examine the relationships among these variables, this study is built upon an integrated theoretical framework that combines the Fear-Avoidance Model and Cognitive-Behavioural Theory. The Fear-Avoidance Model [29] provides the core explanatory pathway for maladaptive responses to symptoms, proposing that a catastrophic misinterpretation of bodily sensations (e.g., AF symptoms) elicits fear, which subsequently drives persistent avoidance behaviour. Cognitive-Behavioural Theory [30] complements this by elucidating the cognitive processes through which a specific fear of exercise-induced bodily sensations becomes reinforced and generalised, evolving into a broader, irrational fear of physical activity itself—namely, kinesiophobia. The integration of these two theories offers a comprehensive, stepwise framework for understanding the psychological transition from symptom perception to pervasive activity avoidance: specifically, from catastrophic appraisal of symptoms to fear of bodily sensations (exercise sensitivity), and further to a generalised fear of movement (kinesiophobia). Based on this integrated theoretical framework, the present study examines the chain-mediating roles of exercise sensitivity and kinesiophobia in the relationship between symptom burden and physical activity among AF patients. The following hypotheses are proposed:

H1: Symptom burden is hypothesized to be directly and negatively associated with physical activity.
H2: Exercise sensitivity is a mediator in the relationship between symptom burden and physical activity.
H3: Kinesiophobia is a mediator in the relationship between symptom burden and physical activity.
H4: Exercise sensitivity and kinesiophobia function as sequential mediators in the statistical model of the relationship between symptom burden and physical activity.

**Fig 1:**
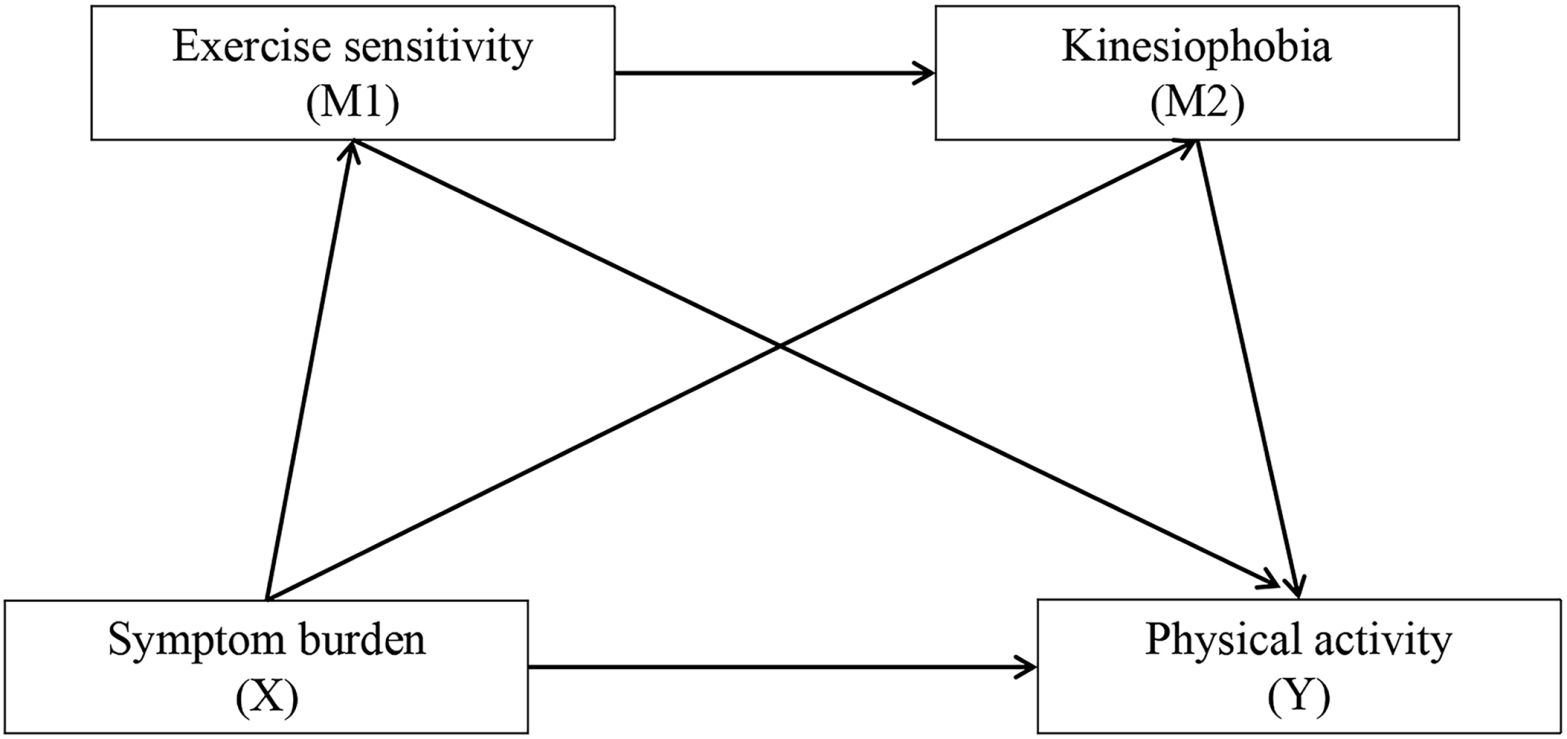
Conceptual model.

The hypothesised chain-mediation model is illustrated in Fig 1. Confirmation of this model would not only advance our theoretical understanding of the psychological mechanisms contributing to physical inactivity in AF patients but also provide a direct, actionable basis for designing targeted interventions to address exercise sensitivity and kinesiophobia in clinical practice.

## 2. Methods

### 2.1. Study design and participants

This study adopted a cross-sectional design. Between April and October 2024, patients with AF were recruited using a convenience sampling method from the cardiology departments of five tertiary hospitals in Chongqing, China: The First Affiliated Hospital of Chongqing Medical University, University Town Hospital of Chongqing Medical University, The Ninth People’s Hospital of Chongqing, Yubei District People’s Hospital, and Changshou District People’s Hospital. The inclusion criteria were: (i) aged 18 years or older; (ii) a confirmed diagnosis of AF [8]; (iii) classified as New York Heart Association class I–III; (iv) conscious and able to complete questionnaires independently or with researcher assistance; (v) independent ambulation ability; and (vi) provision of written informed consent prior to the survey. The exclusion criteria were: (i) a history of ablation for AF (including radiofrequency, cryoballoon, pulsed field, and surgical ablation); (ii) contraindications to exercise (e.g., severe osteoarticular disorders, acute myocardial infarction); or (iii) significant mental, speech, or cognitive impairment. This was defined as either a documented medical diagnosis in the patient’s chart that would impair their ability to participate meaningfully (e.g., dementia, Alzheimer’s disease, aphasia, severe uncontrolled psychiatric disorder such as schizophrenia or bipolar disorder), or a clinical judgment by the attending physician or the trained researcher that the patient’s cognitive or communicative status would prevent them from providing reliable responses, even with researcher assistance.

### 2.2. Measures

#### 2.2.1. Sociodemographic and clinical characteristics.

A structured questionnaire was used to collect data on sex, age, body mass index (BMI), educational level, occupation type, marital status, residential pattern, average monthly household income, comorbidities (e.g., heart failure, hypertension, diabetes, ischaemic heart disease, chronic obstructive pulmonary disease (COPD), anxiety), resting heart rate (RHR), AF duration, and AF type. A comorbidity was considered present if a corresponding diagnosis was reported by the patient as having been made by a specialist physician; for uncertain cases, the diagnosis was verified through on‑site medical record review. AF type was classified according to current guidelines as paroxysmal (episodes that terminate spontaneously or with intervention within 7 days), persistent (continuous AF lasting > 7 days), long-standing persistent (continuous AF lasting > 12 months), or permanent (AF accepted by patient and physician, with no further rhythm control interventions planned) [8]. Relevant variables from this dataset were treated as control variables in the subsequent analysis.

#### 2.2.2. Symptom burden.

Symptom burden was assessed using the symptom subscale of the University of Toronto Atrial Fibrillation Severity Scale (AFSS) [31]. This 7-item subscale evaluates the frequency of core AF-related symptoms over the preceding four weeks on a 6-point Likert scale (0 = “no symptom” to 5 = “a great many symptoms”). Total scores range from 0 to 35, with higher scores indicating greater symptom frequency and burden. In this study, we employed the Chinese version of the symptom subscale of the AFSS, which was validated by Ge et al. [32], which showed good internal consistency (Cronbach’s α = 0.835).

#### 2.2.3. Exercise sensitivity.

Exercise sensitivity was measured using the Exercise Sensitivity Questionnaire (ESQ) [18]. This 18-item instrument assesses fear elicited by bodily sensations during physical exertion. The scale assesses relatively stable, trait-like fear of bodily sensations during exertion and does not refer to a specific retrospective time window. Each item is scored on a 5-point Likert scale ranging from 0 (“not at all”) to 4 (“always”). Total scores range from 0 to 72, with higher scores indicating greater fear of bodily sensations during activity. In this study, we employed the Chinese version of the ESQ translated and validated by Wang et al. [33]. In their validation study of 499 hospitalized cardiovascular patients, the Chinese ESQ demonstrated good construct validity (confirmatory factor analysis: CFI = 0.978, RMSEA = 0.056) and test-retest reliability (0.932) [33]. In our sample of AF patients, the ESQ showed excellent internal consistency (Cronbach’s α = 0.972).

#### 2.2.4. Kinesiophobia.

Kinesiophobia was measured using the Tampa Scale for Kinesiophobia Heart (TSK-SV Heart) [27]. This 17-item scale comprises four dimensions: “perceived risk,” “avoidance of exercise,” “fear of injury,” and “decreased self-function”. The scale measures a generalised and relatively stable fear of movement without reference to a specific retrospective period. Each item is scored on a 4-point Likert scale from 1 (“strongly disagree”) to 4 (“strongly agree”), with items 4, 8, 12, and 16 being reverse-scored. Total scores range from 17 to 68, with higher scores indicating greater kinesiophobia. This study used the Chinese version of the TSK-SV Heart validated by Lei et al. [34], which displayed good internal consistency (Cronbach’s α = 0.855).

#### 2.2.5. Physical activity.

Physical activity was assessed using the short form of the International Physical Activity Questionnaire (IPAQ-SF) [35]. The IPAQ-SF records the frequency (days/week) and duration (minutes/day) of walking, moderate-intensity, and vigorous-intensity activities in the previous week. Metabolic equivalent of task (MET) values were assigned as follows: walking = 3.3 METs, moderate activity = 4.0 METs, vigorous activity = 8.0 METs. Weekly activity volume for each category was calculated as MET-minutes (MET value x minutes/day x days/week). Total weekly physical activity was computed as the sum of MET-minutes across all three categories. In this study, we employed the Chinese version of the IPAQ-SF validated by Qu et al. [36], which showed excellent reliability and validity.

### 2.3. Data collection and ethical approval

The study was conducted in accordance with the Declaration of Helsinki and was approved by the Ethics Committee of the First Affiliated Hospital of Chongqing Medical University (Approval No. 2024–161–01) in March 2024. Prior to data collection, trained researchers explained the study purpose and procedures, assured participants of confidentiality and their right to withdraw, and obtained written informed consent. Data collection was adapted to participant ability: most completed the questionnaires independently via an electronic survey platform with researcher support available; for those unable to do so, researchers conducted face-to-face interviews and recorded responses verbatim. All questionnaires were checked for completeness on-site. To reduce potential common method variance, all questionnaires were administered anonymously, and the TSK‑SV Heart includes reverse‑coded items. To ensure confidentiality, all data were anonymised and de‑identified throughout the study.

### 2.4. Statistical analysis

Data were analysed using SPSS 25.0. As the data were not normally distributed, continuous variables are presented as median with 25th and 75th percentiles [*M* (*p*25, *p*75)], and categorical variables as frequency and percentage. Common method variance (CMV) was assessed using Harman’s single-factor test and unmeasured latent method construct (ULMC). Differences in physical inactivity across patient characteristics were examined using the Mann-Whitney *U* test or the chi-square test, as appropriate. Relationships among symptom burden, exercise sensitivity, kinesiophobia, and physical activity were examined using Spearman’s rank correlation analysis. Covariates for the mediation analyses were selected based on univariate analyses of physical inactivity: variables significant at α = 0.05 were retained. For the subgroup analyses, univariate analyses were conducted within each stratum, and the same selection criterion was applied. Categorical variables entered in the mediation models were coded as follows: sex (1 = male, 2 = female); educational level (1 = junior high school or below, 2 = high school or above); marital status (1 = never married/separated/divorced/widowed, 2 = married/cohabitating); average monthly household income (1 = < 3000 Yuan, 2 = 3000–6000 Yuan, 3 = > 6000 Yuan); AF duration (1 = < 3 years, 2 = 3–5 years, 3 = > 5 years); AF type (1 = permanent, 2 = long-standing persistent, 3 = persistent, 4 = paroxysmal). Comorbidities (heart failure, diabetes, ischaemic heart disease, chronic obstructive pulmonary disease, anxiety) were coded as 1 = yes, 2 = no. The chain-mediating effects were tested using the PROCESS macro (version 3.5, Model 6) with 5,000 bias‑corrected bootstrap resamples and 95% confidence intervals. The mediator order was specified as symptom burden (X)→exercise sensitivity (M₁)→kinesiophobia (M₂)→physical activity (Y). Collinearity was assessed using variance inflation factors (VIFs) for all predictors in each regression model. A sensitivity analysis was performed using log‑transformed physical activity as the outcome. The significance level was set at *α = 0.05*.

## 3. Results

### 3.1. Common method variance

CMV was assessed using two approaches. First, as a preliminary screen, Harman’s single‑factor test was conducted. An unrotated exploratory factor analysis of all 42 measurement items yielded nine factors with eigenvalues greater than 1, which together accounted for 61.75% of the total variance. The first factor explained 31.48% of the variance. However, as demonstrated by Howard et al. [37], this test has limited sensitivity and is not a reliable method for detecting CMV. Consequently, the ULMC approach was employed as a more rigorous method. A baseline multi‑trait confirmatory factor analysis model (M1) was specified, after which a second model (M2) was constructed by adding a common method factor on which all scale items were allowed to load in addition to their intended construct factors. The changes in fit indices between M1 and M2 were minimal: Δχ²/df = 0.036, ΔRMSEA = 0.001, ΔIFI = 0.003, ΔTLI = 0.001, and ΔCFI = 0.003, all well below conventional thresholds for the presence of meaningful method variance. These negligible improvements indicate that the addition of a common method factor did not substantially improve model fit. Taken together, the results of these analyses suggest that serious common method variance is unlikely to be present in this study.

### 3.2. Participant characteristics and univariate analysis

A total of 653 eligible patients were approached during the study period. Of these, 597 agreed to participate and completed the survey. After stringent screening, 38 questionnaires were excluded due to completion time < 600 seconds, 16 were removed for logical errors, and 10 were discarded due to missing critical data, leaving 533 valid responses for final analysis, yielding an overall effective response rate of 81.6% (533/653). Among the 56 patients who declined participation, the primary reasons were: lack of interest (n = 33), time constraints (n = 13), and feeling unwell on the day of the survey (n = 10). The sample included 297 (55.7%) male and 236 (44.3%) female participants. Physical inactivity was identified in 414 patients (77.7%). Univariate analysis identified several factors significantly associated with physical inactivity in AF patients: sex, age, educational level, marital status, average monthly household income, heart failure, diabetes, ischaemic heart disease, COPD, AF duration, AF type, symptom burden, exercise sensitivity, and kinesiophobia. Detailed results are presented in Table 1.

**Table 1:** Univariate Analysis of Physical Inactivity in AF Patients (n = 533).

Variable	Total Participants (n = 533)	Physical Inactivity		χ^2^/Z	p
		Yes (n = 414)	No (n = 119)		
**Sex** (n [%])				22.576^a^	< 0.001
Male	297 (55.7)	208 (50.2)	89 (74.8)		
Female	236 (44.3)	206 (49.8)	30 (25.2)		
**Age** (years, *M* [*p*25, *p*75])	71 (63, 78)	73 (66, 79)	62 (57, 72)	−6.929^b^	< 0.001
**BMI** (kg/m², *M* [*p*25, *p*75])	24.0 (22.1, 26.3)	24.1 (22.0, 26.4)	23.9 (22.4, 25.6)	−0.442^b^	0.658
Educational level (n [%])				16.663^a^	< 0.001
Junior high school or below	360 (67.5)	298 (72.0)	62 (52.1)		
High school or above	173 (32.5)	116 (28.0)	57 (47.9)		
Occupation type (n [%])				2.552^a^	0.279
Physical labor	263 (49.4)	203 (49.0)	60 (50.4)		
Mental labor	224 (42.0)	171 (41.3)	53 (44.5)		
Unemployed	46 (8.6)	40 (9.7)	6 (5.0)		
Marital status (n [%])				10.963^a^	0.001
Married/cohabitating	456 (85.6)	343 (82.9)	113 (95.0)		
Never married/separated /divorced/widowed	77 (14.4)	71 (17.1)	6 (5.0)		
Residential pattern (n [%])				1.585^a^	0.208
Living alone	36 (6.8)	31 (7.5)	5 (4.2)		
Living with others	497 (93.2)	383 (92.5)	114 (95.8)		
Average monthly household **income** (Yuan, n [%])				14.342^a^	0.001
< 3000	139 (26.1)	120 (29.0)	19 (16.0)		
3000–6000	281 (52.7)	219 (52.9)	62 (52.1)		
> 6000	113 (21.2)	75 (18.1)	38 (31.9)		
Heart failure (n [%])				62.978^a^	< 0.001
Yes	237 (44.5)	222 (53.6)	15 (12.6)		
No	296 (55.5)	192 (46.4)	104 (87.4)		
Hypertension (n [%])				0.397^a^	0.529
Yes	309 (58.0)	243 (58.7)	66 (55.5)		
No	224 (42.0)	171 (41.3)	53 (44.5)		
Diabetes (n [%])				6.279^a^	0.012
Yes	111 (20.8)	96 (23.2)	15 (12.6)		
No	422 (79.2)	318 (76.8)	104 (87.4)		
Ischaemic heart disease (n [%])				27.189^a^	< 0.001
Yes	213 (40.0)	190 (45.9)	23 (19.3)		
No	320 (60.0)	224 (54.1)	96 (80.7)		
**COPD** (n [%])				5.118^a^	0.024
Yes	45 (8.4)	41 (9.9)	4 (3.4)		
No	488 (91.6)	373 (90.1)	115 (96.6)		
**Anxiety**				0.410^a^	0.522
Yes	91 (17.1)	73 (17.6)	18 (15.1)		
No	442 (82.9)	341 (82.4)	101 (84.9)		
**RHR** (beats/min, n [%])				5.668^a^	0.059
< 60	58 (10.9)	40 (9.7)	18 (15.1)		
60–90	370 (69.4)	285 (68.8)	85 (71.4)		
> 90	105 (19.7)	89 (21.5)	16 (13.4)		
**AF duration** (years, n [%])				12.757^a^	0.002
< 3	300 (56.3)	216 (52.2)	84 (70.6)		
3–5	97 (18.2)	82 (19.8)	15 (12.6)		
> 5	136 (25.5)	116 (28.0)	20 (16.8)		
**AF type** (n [%])				14.626^a^	0.002
Paroxysmal AF	231 (43.3)	162 (39.1)	69 (58.0)		
Persistent AF	212 (39.8)	174 (42.0)	38 (31.9)		
Long-standing persistent AF	62 (11.6)	55 (13.3)	7 (5.9)		
Permanent AF	28 (5.2)	23 (5.6)	5 (4.2)		
**Symptom subscale of AFSS** (scores, *M* [*p*25, *p*75])	14 (8, 20)	15.5 (10, 21)	7 (5, 13)	−8.978^b^	< 0.001
**ESQ** (scores, *M* [*p*25, *p*75])	46 (28, 66)	56 (32, 69)	29 (24, 37)	−8.858^b^	< 0.001
**TSK**-**SV Heart** (scores, *M* [*p*25, *p*75])	44 (36, 50)	47 (38, 51)	34 (32, 38)	−11.070^b^	< 0.001

**Abbreviations:** AF, atrial fibrillation; BMI, body mass index; COPD, Chronic obstructive pulmonary disease; RHR, Resting heart rate;

AFSS, University of Toronto Atrial Fibrillation Severity Scale; ESQ, Exercise Sensitivity Questionnaire; TSK-SV Heart, Tampa Scale for Kinesiophobia Heart.

**Note:** ^a^ *χ*^2^; ^b^ Z.

### 3.3. Correlations among main variables

Spearman’s correlation analysis revealed that symptom burden was positively correlated with exercise sensitivity (r = 0.551, p < 0.01) and kinesiophobia (r = 0.633, p < 0.01), and negatively correlated with physical activity (r = −0.612, p < 0.01). Exercise sensitivity was also positively correlated with kinesiophobia (r = 0.775, p < 0.01) and negatively correlated with physical activity (r = −0.629, p < 0.01). Furthermore, kinesiophobia was negatively correlated with physical activity (r = −0.685, p < 0.01). All correlation coefficients are shown in Table 2.

**Table 2:** Medians and correlation analysis of variables (n = 533).

Variable	Median (*M* [*p*25, *p*75])	Symptom burden	Exercise sensitivity	Kinesiophobia
Symptom burden	14 (8, 20)	1		
Exercise sensitivity	46 (28, 66)	0.551^**^	1	
Kinesiophobia	44 (36, 50)	0.633^**^	0.775^**^	1
Physical activity	1617 (809, 2772)	−0.612^**^	−0.629^**^	−0.685^**^

**Note:** ^**^ *p* < 0.01.

### 3.4. Chain-mediation analysis

A chain-mediation model was performed using Hayes’ PROCESS macro, controlling for sex, age, educational level, marital status, average monthly household income, heart failure, diabetes, ischaemic heart disease, COPD, AF duration, and AF type. Regression analysis showed that symptom burden was significantly positively associated with both exercise sensitivity (β = 1.211, *p* < 0.001) and kinesiophobia (β = 0.267, *p* < 0.001). Furthermore, symptom burden, exercise sensitivity, and kinesiophobia were all significantly negatively associated with physical activity (β = −27.633, β = −17.018, and β = −45.156, respectively; all *p* < 0.001). VIFs were below 3.5 in all models, indicating no serious multicollinearity. Complete results are provided in Table 3.

**Table 3:** Regression analysis results of chain-mediation model.

Variable	Model 1		Model 2		Model 3	
β	t	β	t	β	t
Constants	60.204	5.509^***^	29.331	8.372^***^	6353.092	6.984^***^
Sex	8.737	6.346^***^	1.050	2.357^*^	−50.911	−0.467
Age	−0.102	−1.467	−0.030	−1.378	−22.632	−4.258^***^
Education level	−4.932	−3.000^**^	−0.977	−1.890	−58.994	−0.467
Marital status	−7.850	−3.920^***^	0.734	1.159	−3.194	−0.021
Average monthly household income	−1.444	−1.279	−0.443	−1.257	14.163	0.165
Heart failure	0.403	0.252	−0.999	−2.004^*^	464.442	3.808^***^
Diabetes	−0.230	−0.139	1.243	2.419^*^	1.962	0.016
Ischaemic heart disease	0.937	0.655	−0.092	−0.206	−20.212	−0.186
COPD	−3.083	−1.245	−0.359	−0.464	81.394	0.432
AF duration	−1.461	−1.723	0.489	1.846	−16.678	−0.257
AF type	−1.461	−1.670	−0.500	−1.829	−95.694	−1.432
Symptom burden	1.211	10.386^***^	0.267	6.698^***^	−27.633	−2.724^***^
Exercise sensitivity			0.263	19.208^***^	−17.018	−3.906^***^
Kinesiophobia					−45.156	−4.221^***^
R	0.637		0.835		0.663	
R^2^	0.406		0.697		0.439	
F	29.650		91.880		29.008	

**Abbreviations**: AF, atrial fibrillation; COPD, chronic obstructive pulmonary disease.

**Note**: Model 1: Independent Variable: Symptom burden; Dependent Variable: Exercise sensitivity.

Model 2: Independent Variable: Symptom burden, Exercise sensitivity; Dependent Variable: Kinesiophobia.

Model 3: Independent Variable: Symptom burden, Exercise sensitivity, Kinesiophobia; Dependent Variable: Physical activity.

Sex was coded as 1 = male, 2 = female. Heart failure was coded as 1 = yes, 2 = no.

*** *p* < 0.001; ** *p* < 0.01; * *p* < 0.05.

**Fig 2:**
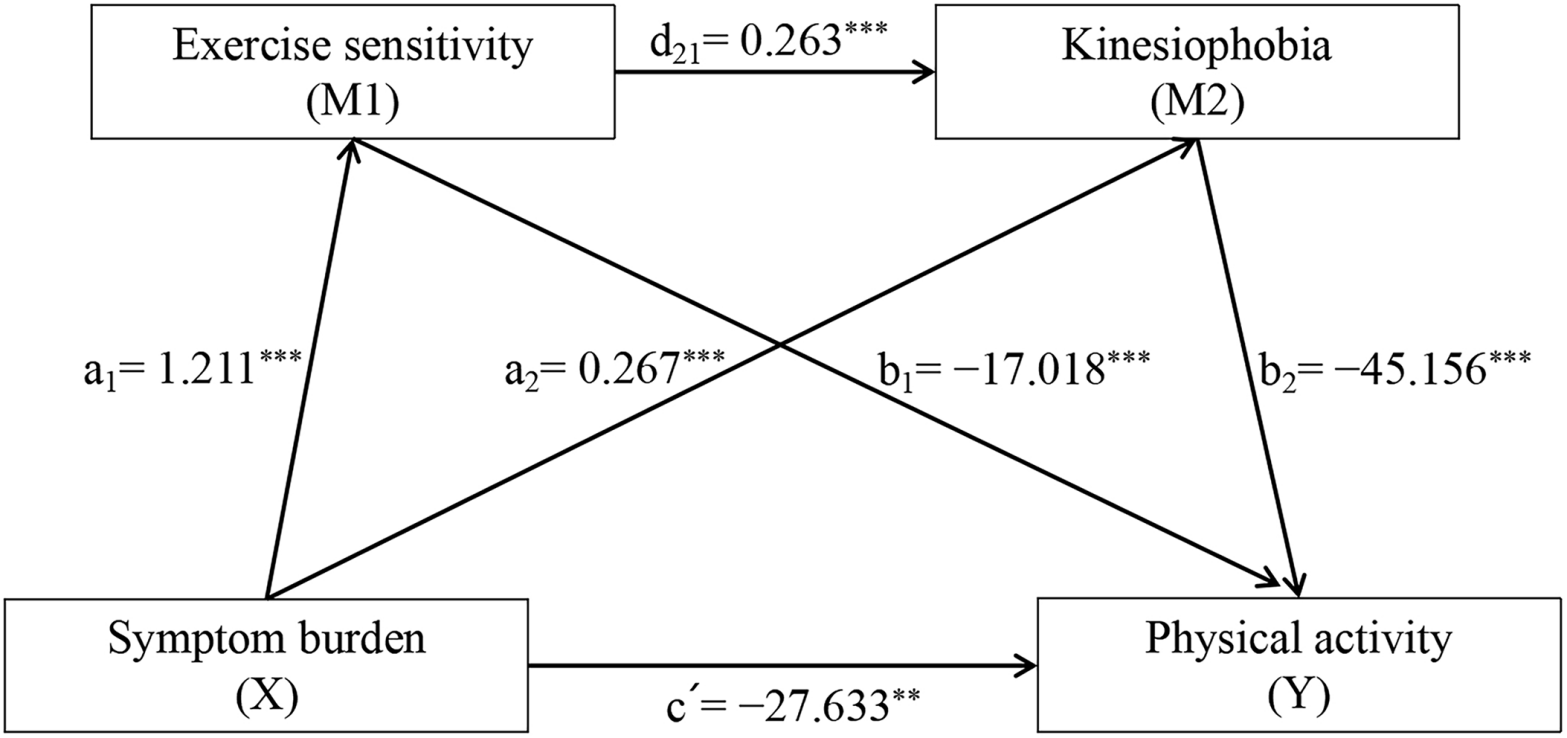
Chain-mediation model. Note: ^***^p < 0.001, ^**^p < 0.01.

Mediation effects were tested using the bootstrap method with 5,000 resamples. The results indicated that the indirect effects were statistically significant, as the 95% confidence intervals for both the total and all specific indirect effects excluded zero. Three significant indirect pathways were identified: (1) Symptom burden→exercise sensitivity→physical activity (indirect effect = −20.608, accounting for 27.60% of the total effect); (2) Symptom burden→kinesiophobia→physical activity (indirect effect = −12.075, accounting for 16.17% of the total effect); (3) Symptom burden→exercise sensitivity→kinesiophobia→physical activity (indirect effect = −14.354, accounting for 19.22% of the total effect). The total effect of symptom burden on physical activity was −74.670, comprising a direct effect of −27.633 (37.00% of the total effect) and a total indirect effect of −47.037 (62.99% of the total effect). In addition, a sensitivity analysis using log‑transformed physical activity yielded consistent results for all indirect pathways. Detailed results are presented in Table 4 and illustrated in Fig 2.

**Table 4:** Path analysis results.

Influence Pathway	β	BootSE	BootCI	Proportion of Total Effect
Symptom burden→Exercise sensitivity →Physical activity	−20.608	8.187	[−37.890, −6.019]	27.60%
Symptom burden→Kinesiophobia→Physical activity	−12.075	4.608	[−21.822, −3.754]	16.17%
Symptom burden→Exercise sensitivity→ Kinesiophobia→Physical activity	−14.354	4.970	[−24.251, −4.590]	19.22%
Total Effect	−74.670	9.599	[−93.528, −55.813]	
Direct Effect	−27.633	10.143	[−47.559, −7.708]	37.00%
Total Indirect Effect	−47.037	7.062	[−62.027, −34.706]	62.99%

**Note**: All confidence intervals not containing zero indicate statistically significant effects at α = 0.05. β represents unstandardized coefficients.

To examine whether the proposed mediation model differed across patient subgroups, we conducted additional analyses stratified by heart failure status. The results are presented in Table 5 (patients without heart failure, n = 296) and Table 6 (patients with heart failure, n = 237). In the subgroup without heart failure, adjusted for sex, age, educational level, marital status, diabetes, ischaemic heart disease, and anxiety, the total indirect effect remained statistically significant, accounting for 50.09% of the total effect. However, the pattern of specific indirect effects differed from the full sample: while the pathways via exercise sensitivity alone (symptom burden→exercise sensitivity→physical activity) and the sequential pathway (symptom burden→exercise sensitivity→kinesiophobia→physical activity) remained significant, the pathway via kinesiophobia alone (symptom burden→kinesiophobia→physical activity) was no longer statistically significant (95% CI: −21.712 to 0.112). The direct effect remained significant. In the subgroup with heart failure, adjusted for sex, age, and average monthly household income, all three indirect pathways were statistically significant, with the total indirect effect accounting for 78.11% of the total effect. The direct effect was not statistically significant in this subgroup (95% CI: −32.679 to 3.714).

**Table 5:** Path analysis results for patients without heart failure (n = 296).

Influence Pathway	β	BootSE	BootCI	Proportion of Total Effect
Symptom burden→Exercise sensitivity →Physical activity	−20.694	10.773	[−45.302, −3.229]	24.56%
Symptom burden→Kinesiophobia→Physical activity	−8.957	5.636	[−21.712, 0.112]	10.63%
Symptom burden→Exercise sensitivity→ Kinesiophobia→Physical activity	−12.558	6.554	[−25.448, −0.100]	14.90%
Total Effect	−84.269	16.628	[−116.998, −51.541]	
Direct Effect	−42.060	16.145	[−73.839, −10.281]	49.91%
Total Indirect Effect	−42.209	10.770	[−65.345, −23.742]	50.09%

**Note**: All confidence intervals not containing zero indicate statistically significant effects at α = 0.05. β represents unstandardized coefficients.

**Table 6:** Path analysis results for patients with heart failure (n = 237).

Influence Pathway	β	BootSE	BootCI	Proportion of Total Effect
Symptom burden→Exercise sensitivity →Physical activity	−17.969	6.109	[−31.548, −7.093]	27.16%
Symptom burden→Kinesiophobia→Physical activity	−18.577	5.382	[−29.856, −8.657]	28.08%
Symptom burden→Exercise sensitivity→ Kinesiophobia→Physical activity	−15.134	3.994	[−23.414, −7.860]	22.87%
Total Effect	−66.162	8.480	[−82.870, −49.454]	
Direct Effect	−14.482	9.235	[−32.679, 3.714]	21.89%
Total Indirect Effect	−51.680	6.841	[−65.443, −38.833]	78.11%

**Note**: All confidence intervals not containing zero indicate statistically significant effects at α = 0.05. β represents unstandardized coefficients.

## 4. Discussion

Drawing on an integrated framework that combines the fear-avoidance model and cognitive behavioural theory, this study employed a chain-mediation model to explore the psychological mechanisms linking symptom burden to physical activity in AF patients. The results indicate that symptom burden was not only directly and negatively associated with physical activity—accounting for 37.0% of the total effect—but also indirectly associated with it via three pathways: the independent mediation of exercise sensitivity (27.6%), the independent mediation of kinesiophobia (16.2%), and their sequential chain mediation (19.2%). Overall, the total indirect effect accounted for 62.99% of the total effect. This large proportion is consistent with the hypothesis that psychological factors play a substantial role in explaining the statistical association between higher symptom burden and reduced physical activity levels in this sample. However, due to the cross-sectional design, the precise nature and magnitude of their causal contribution cannot be definitively determined from this study alone. These findings underscore that AF management should extend beyond symptom control to also include interventions addressing patients’ cognitive and emotional responses.

### 4.1. The relationship between symptom burden and physical activity

The findings are consistent with Hypothesis 1, indicating a significant direct negative association between symptom burden and physical activity. This aligns with previous reports that AF patients with a greater symptom burden experience reduced physical activity [14]. Common AF symptoms—including dyspnoea, palpitations, fatigue, exercise intolerance, dizziness, and chest pain—directly constrain patients’ exercise capacity [38]. Recent evidence provides mechanistic support for this direct pathway. Studies have shown that patients with AF exhibit significantly higher myocardial oxygen extraction compared to those in sinus rhythm, reflecting reduced cardiac metabolic efficiency that is directly associated with dyspnea, exercise intolerance, and palpitations during physical activity [39]. This finding suggests that the symptoms limiting physical activity in AF have a measurable physiological substrate.

### 4.2. The mediating role of exercise sensitivity

Hypothesis 2 was supported, indicating that exercise sensitivity functions as a mediator in the relationship between symptom burden and physical activity. AF symptoms such as palpitations, dyspnoea, and dizziness closely resemble normal physiological responses to exertion. Patients with high symptom burden are therefore prone to catastrophically misinterpreting these exercise‑related sensations as signals of AF onset or cardiac decompensation [18]. This catastrophic appraisal generates fear of the bodily sensations themselves—exercise sensitivity [17,18]—which, in turn, drives avoidance of physical activity [17,40]. Notably, this pathway accounted for the largest proportion of the indirect effect, highlighting fear of exercise‑related sensations as a primary psychological mechanism through which symptoms translate into avoidance behaviour. Consequently, routine screening for exercise sensitivity in cardiac rehabilitation is warranted. For affected individuals, interventions such as interoceptive exposure based on cognitive-behavioural therapy [41] could help patients safely re-associate bodily sensations with safety, thereby promoting increased activity.

### 4.3. The mediating role of kinesiophobia

Consistent with Hypothesis 3, kinesiophobia was found to function as an independent mediator in the relationship between symptom burden and physical activity—a finding consistent with previous research [16]. Patients with high symptom burden often develop a pervasive and irrational fear of physical exertion, primarily driven by concerns that exercise may trigger or worsen their symptoms [42]. This fear not only correlates positively with symptom burden [24] but also substantially impedes both the initiation and maintenance of exercise, thereby contributing to reduced physical activity [23,43]. The mediating role of kinesiophobia underscores that exercise management in AF should extend beyond symptom control to also address symptom-related fear. Integrating relaxation techniques (e.g., mindfulness meditation, guided imagery) [44] with cognitive interventions grounded in social cognitive theory may help alleviate kinesiophobia [45] and, in turn, foster greater adherence to and engagement in physical activity.

Subgroup analyses revealed that the relationship between symptom burden and physical activity differed by heart failure status. In AF patients without heart failure, symptom burden was directly associated with physical activity and indirectly associated via exercise sensitivity, whereas the independent pathway through kinesiophobia was not statistically significant. In AF patients with heart failure, all three indirect pathways were significant, and the direct association was not. These patterns suggest that the mediating mechanisms may vary with comorbidity burden rather than applying uniformly across all patients. In both subgroups, the sequential chain‑mediation pathway was significant, consistent with the possibility that fear generalises from specific bodily sensations to a broader fear of movement regardless of heart failure status. The key divergence concerns whether kinesiophobia is associated with symptom burden independently of exercise sensitivity. In patients without heart failure, the non‑significant independent kinesiophobia pathway suggests that, in this subgroup, kinesiophobia may be associated with symptom burden predominantly through exercise sensitivity. In patients with heart failure, the significant independent pathway suggests that symptom burden may also be associated with kinesiophobia without this intermediate step. This interpretation is consistent with prior research reporting that kinesiophobia is linked to symptom burden in heart failure patients [46] and that psychological factors are associated with activity behaviour in this population [47].

### 4.4. The chain-mediation effect of exercise sensitivity and kinesiophobia

Our findings are consistent with Hypothesis 4, revealing a significant chain-mediation pattern in the data: symptom burden→exercise sensitivity→kinesiophobia→physical activity. This result is consistent with a “fear generalisation” process, in which the psychological impact of symptoms evolves from a specific fear of bodily sensations into a broader fear of movement itself. Notably, this sequential pathway accounted for 19.2% of the total effect, underscoring its distinct and substantial role in translating symptom burden into behavioural avoidance. Our observed pathway illustrates a core cognitive-behavioral sequence. Informed by the fear-avoidance model [29], the process originates when patients catastrophically misinterpret benign, exercise-induced bodily sensations (e.g., elevated heart rate)—which closely resemble AF symptoms—as signs of danger, thereby fostering a specific fear of these sensations (exercise sensitivity). Cognitive-behavioural theory [30] explains the subsequent progression: this focused fear becomes cognitively embedded as an automatic negative association (“exercise = threat”), leading to a catastrophic appraisal of physical activity itself and the development of a generalised fear of movement (kinesiophobia), ultimately resulting in activity avoidance. This stepwise ‘misinterpretation→specific fear→generalised fear→avoidance’ mechanism empirically clarifies how fear generalises in AF, extending prior evidence that exercise sensitivity predicts kinesiophobia [25]. The sequential nature of this mediation suggests the potential value of a stepped clinical approach. First, interventions should aim to reduce exercise sensitivity through cognitive restructuring and interoceptive exposure, targeting the initial misinterpretation of bodily signals. Subsequently, efforts should address the resultant kinesiophobia using relaxation techniques (e.g., mindfulness, guided imagery) combined with cognitive reappraisal to mitigate the generalised fear of exercise. Such a sequential strategy may help systematically disrupt the “symptom–fear–avoidance” cycle and promote sustained engagement in physical activity among patients with AF.

### 4.5. Strengths and limitations

This study has several strengths. First, based on an integrated theoretical framework, it proposed and tested a chain-mediation model (“exercise sensitivity→kinesiophobia”) that explains how symptom burden affects physical activity in AF, offering a novel theoretical perspective. Second, the identified pathway provides empirical support for designing stepped psychological-behavioural interventions. Third, the use of well-validated instruments to assess all key constructs enhances the reliability and validity of the findings. Several limitations must be acknowledged. First, the cross‑sectional design precludes causal inferences. In the chain-mediation analysis, exercise sensitivity was modelled simultaneously as an outcome of symptom burden and a predictor of kinesiophobia (Model 2), and all three predictors were entered together in the equation for physical activity (Model 3). This specification assumes a temporal ordering—symptom burden→exercise sensitivity→kinesiophobia→physical activity—that cannot be verified with cross‑sectional data. Our results therefore indicate only that the observed associations are consistent with this theoretically derived sequence, not that the variables unfold in this order over time. This limitation is compounded by the different recall windows of the instruments: the AFSS assessed symptoms over the preceding four weeks, whereas the IPAQ‑SF assessed physical activity over the past week. Although the one‑week window is nested within the four‑week window and symptom burden in chronic AF tends to be relatively stable, this temporal mismatch introduces ambiguity. The ESQ and TSK‑SV Heart, which assess relatively stable psychological propensities, do not eliminate this concern. Although exercise sensitivity and kinesiophobia were highly correlated (r = 0.775), variance inflation factors were below 3.5, indicating no serious multicollinearity. Future research using structural equation modeling with latent variables could further examine their discriminant validity. The terms “mediator,” “total effect,” “direct effect,” and “indirect effect” are used in a statistical sense only. Longitudinal or experimental studies with harmonised measurement intervals are needed to confirm the directional hypotheses. Second, symptom burden was assessed using a self-report instrument. Without objective cardiac function measures (e.g., echocardiography, cardiopulmonary exercise testing), we cannot fully disentangle the direct physiological limits on activity from the psychological responses to those limits, nor determine the extent to which reported symptoms reflect AF-specific pathology versus comorbid conditions. Future studies should incorporate such objective measures, along with data on medication profiles and heart rhythm at the time of assessment, to better distinguish physiological from psychological pathways. Third, all core variables were measured using self‑report questionnaires at a single time point, which may introduce common method variance. Although the procedural controls and the ULMC results provided converging evidence that serious CMV is unlikely, as Howard et al. [37] have demonstrated, all post‑hoc CMV tests have limitations. Future studies should incorporate marker‑variable techniques or multi‑method designs for more rigorous assessment. Fourth, physical activity was assessed using the self-reported IPAQ-SF, which may overestimate activity levels compared to objective measures. We conducted a sensitivity analysis using log-transformed physical activity, which confirmed the consistency of the path estimates. We did not conduct additional sensitivity analyses using alternative estimators (e.g., dichotomous outcome, robust standard errors, or quantile regression). Future studies should incorporate objective measures such as accelerometry for more precise assessment. Fifth, the use of convenience sampling from five hospitals in a single city may limit generalizability. The physical inactivity rate in our sample (77.7%) was higher than the 67.4% reported in a large population‑based AF cohort [9], likely reflecting the older age (median 71 years) and high comorbidity burden (44.5% with heart failure, 40.0% with ischaemic heart disease) of our hospital‑based sample. Future research should test the model in broader populations. Sixth, our subgroup analyses suggested that the mediating mechanisms may differ by heart failure status, but several limitations qualify these findings. Sample size was insufficient for comprehensive subgroup analyses across multiple comorbidity profiles. Covariates were selected based on univariate analyses within each stratum and therefore differ from those in the full-sample model; consequently, the total effects across models are not directly comparable. Heart failure status was based on patient self‑report of a physician‑confirmed diagnosis, without systematic assessment of ejection fraction or heart failure subtype, which may have introduced misclassification. Other comorbidities—including hypertension, ischaemic heart disease, and diabetes—may also moderate the mediating pathways, as may broader social determinants of health such as neighborhood deprivation [48], and these were not examined. Similarly, AF type (paroxysmal vs. persistent/permanent) was not examined as a potential moderator, although its role in shaping fear‑avoidance responses warrants investigation. Future studies with larger samples and pre-specified analytic plans should systematically test whether these factors moderate the indirect pathways identified here.

## 5. Conclusions

In summary, grounded in an integrated fear-avoidance and cognitive-behavioural framework, this study provides evidence consistent with a model in which symptom burden in AF patients is associated with reduced physical activity both directly and, more substantially, through its association with exercise sensitivity and kinesiophobia, which function as independent and sequential mediators in this statistical model. These findings advance the theoretical understanding of psychological drivers of inactivity and have important clinical implications. AF care should extend beyond symptom control to routinely assess and address exercise sensitivity and kinesiophobia. Future research should explore whether stepped interventions addressing exercise sensitivity and kinesiophobia can disrupt the “symptom–fear–avoidance” cycle, thereby improving long-term activity levels and health outcomes.

## Supporting information

S1 File De-identified dataset for the cross-sectional study on symptom burden and physical activity in patients with atrial fibrillation. (XLSX)

S2 File Detailed codebook and data dictionary for S1 File. (PDF)
