# Supplementary material for: Influence of symptom burden on physical activity among patients with atrial fibrillation: The chain-mediating roles of exercise sensitivity and kinesiophobia
Source: PLoS One. 2026 Jun 30;21(6):e0352864. doi: 10.1371/journal.pone.0352864 (PMC13318014; doi:10.1371/journal.pone.0352864)
Supplement: S2 File — (PDF) [file pone.0352864.s002.pdf]

Detailed codebook and data dictionary for S1 File

**variable**	**value assignment**
Sex	1=Male, 2=Female
Educational level	1=Junior high school or below, 2=High school or above
Occupation type	1=Unemployed, 2=Mental labor, 3=Physical labor
Marital status	1=Never married/separated/divorced /widowed, 2=Married/cohabitating
Residential pattern	1=Living alone, 2=Living with others
Average monthly household income (Yuan)	1=<3000, 2=3000–6000, 3=>6000
Heart failure	1=Yes, 2=No
Hypertension	1=Yes, 2=No
Diabetes	1=Yes, 2=No
Ischaemic heart disease	1=Yes, 2=No
Chronic obstructive pulmonary disease	1=Yes, 2=No
Anxiety	1=Yes, 2=No
Resting heart rate (beats/min)	1=<60, 2=60–90, 3=>90
Atrial fibrillation duration (years)	1=<3, 2=3–5, 3=>5
Atrial fibrillation type	1=Permanent atrial fibrillation, 2=Long-standing persistent atrial fibrillation, 3=Persistent atrial fibrillation, 4=Paroxysmal atrial fibrillation
